# Preoperative serum pre-albumin as an independent prognostic indicator in patients with localized upper tract urothelial carcinoma after radical nephroureterectomy

**DOI:** 10.18632/oncotarget.13694

**Published:** 2016-11-29

**Authors:** Jiwei Huang, Yanqing Wang, Yichu Yuan, YongHui Chen, Wen Kong, Haige Chen, Jin Zhang, Yiran Huang

**Affiliations:** ^1^ Department of Urology, Ren Ji Hospital, School of Medicine, Shanghai Jiao Tong University, Shanghai, China

**Keywords:** upper tract, urothelial carcinoma, pre-albumin, albumin, prognosis

## Abstract

**Purpose:**

To investigate the prognostic value of preoperative pre-albumin and albumin level in patients with localized upper tract urothelial carcinoma (UTUC) undergoing radical nephroureterectomy.

**Methods and Materials:**

Between January 2003 and June 2013, we evaluated data on 425 patients with nonmetastatic UTUC (Ta-4N0/+M0) who underwent radical nephroureterectomy at our institution. Low pre-albumin level was defined as <20 mg/dl, while hypoalbuminemia was defined as albumin <35 g/L. The associations of preoperative low pre-albumin level and hypoalbuminemia with clinical and pathologic variables were assessed. Univariable and multivariable analyses using the Cox regression model were performed to determine prognostic factors that were associated with cancer specific survival (CSS) and overall survival (OS). The Harrell concordance index with variables only or combined pre-albumin data were used to evaluate the prognostic accuracy.

**Results:**

Compared with patients with high pre-albumin level, patients with low pre-albumin level were more likely to have older age, higher tumor stage, higher rate of diabetes, regional lymph node metastasis and lymphovascular invasion. Meanwhile, hypoalbuminemia was only associated with diabetes. Multivariate analysis identified decreased preoperative pre-albumin level as an independent prognostic factor for CSS (HR 1.85, 95% CI 1.14-3.00, p=0.013) and OS (HR 1.73, 95% CI 1.12-2.70, p=0.015), but not preoperative hypoalbuminemia. The estimated c-index of the multivariate model for CSS and OS increased from 0.771 and 0.760 without pre-albumin to 0.775 and 0.765 when pre-albumin added.

**Conclusions:**

Low preoperative pre-albumin level but not preoperative hypoalbuminemia is a negative independent prognostic factor for survival outcome in patients with UTUC undergoing radical nephroureterectomy.

## INTRODUCTION

Upper tract urothelial carcinoma (UTUC) is an uncommon but aggressive malignant disease that accounts for only 5% to 10% of all urothelial carcinomas [1]. The natural history and oncogenicity of UTUC appear to be rather different from that of urothelial carcinomas of the bladder. Around 50% of UTUCs are muscular invasive at diagnosis compared to only 15% of urothelial carcinomas of the bladder [2, 3]. Although radical nephroureterectomy (RNU) is the surgical standard for the treatment of patients with UTUC, the prognosis for patients who undergo RNU still remains poor. Currently, even after curative therapy, 5-year disease-specific survivals ranging between 50% and 80% have been reported [4, 5].

Pathological T stage, tumor grade, lymph node metastasis, distant metastasis, lymphovascular invasion (LVI) are well established pathological prognostic factors in patients with UTUC [1, 6–7]. Moreover, patient age and tumor multifocality are also independent predictors of patient survival [1, 8]. However, the potential prognostic preoperative factors are still limited in UTUC. Therefore, it is crucial to identify risk factors, which would allow for the assessment of individual risk profiles. The nutritional status of patients with cancer is an important parameter affecting survival outcomes [9, 10].

Pre-albumin, a visceral liver-synthesized protein, is a sensitive marker for determining the state of malnutrition and easily quantified in hospital laboratories. Study has shown that pre-albumin is a remarkable prognostic factor for treatment outcomes of esophagus [11], ovarian [12], and lung cancers [13, 14]. However, the prognostic value of plasma pre-albumin levels for urological cancer has not yet been reported.

Thus, this study was designed to clarify the potential prognostic value of preoperative pre-albumin and albumin level in a large number of patients who have undergone RNU for UTUC.

## RESULTS

### Clinical characteristics

Our final cohort included 279 men (65.6%) and 146 women (34.4%). Patients’ median age at surgery was 67.0 years (interquartile range (IQR), 58.0-75.0) (Table 1). Open RUN was performed in 281 patients (66.1%), while the remaining 144 patients (33.9%) underwent laparoscopic RUN, respectively. Pathological T stage was pTa-1 in 225 cases (52.9%), pT2 in 68 (16.0%), pT3 in 120 (28.2%) and pT4 in 12 (2.8%). Lymphadenectomy was done in 93 patients (21.8%) who presented with clinically enlarged lymph nodes. 86 patients received adjuvant chemotherapy (AC) in this study.

**Table 1:** Clinical and pathological characteristics of 425 UTUC patients stratified according to the presence of low pre-albumin level and hypoalbuminemia

Variables	Pre-Albumin≥20mg/dl	Pre-Albumin<20mg/dl	*P-*value	Albumin≥35g/L	Albumin <35g/L	*P*-value
Age (years), median (IQR)	67.0 (57.8-75.0)	71.0 (60.0-77.0)	0.040	67.0 (58.0-75.0)	72.0 (61.0-77.0)	0.231
Gender, n(%)			0.090			1.000
Male	246(67.2)	33(55.9)		271(66.4)	8(47.1)	
Female	120(32.8)	26(44.1)		137(33.6)	9(52.9)	
Hypertension			0.702			0.488
Yes	121(33.1)	21(35.6)		135(33.1)	7(41.2)	
No	245(66.9)	38(64.4)		273(66.9)	10(58.8)	
Diabetes mellitus			0.049**			0.012**
Yes	41(11.2)	12(20.3)		47(11.5)	6(35.3)	
No	325(88.8)	47(79.7)		361(88.5)	11(64.7)	
Location			0.772			0.787
Renal pelvic	181(49.5)	27(45.8)		201(49.3)	7(41.2)	
Ureteral	118(32.2)	19(32.2)		131(32.1)	6(35.3)	
Multifocal	67(18.3)	13(22.0)		76(18.6)	4(23.5)	
T stage			0.021**			0.137
Ta-1	202(55.2)	23(39.0)		219(53.7)	6(35.3)	
T2-4	164(44.8)	36(61.0)		189(46.3)	11(64.7)	
Lymph node dissection			0.033**			0.381
N0	62(16.9)	7(11.9)		67(16.4)	2(11.8)	
N1-3	16(4.4)	8(13.6)		22(5.4)	2(11.8)	
Nx	288(78.7)	44(74.6)		319(78.2)	13(76.5)	
WHO Grade			0.150			0.154
Low	128(35.0)	15(25.4)		140(34.3)	3(17.6)	
High	238(65.0)	44(74.6)		268(65.7)	14(82.4)	
LVI			0.024**			0.806
Yes	51(13.9)	15(25.4)		63(15.4)	3(17.6)	
No	315(86.1)	44(74.6)		345(84.6)	14(82.4)	
Adjuvant Chemotherapy			0.472			0.758
No	294(80.3)	45(76.3)		326(79.9)	13(76.5)	
Yes	72(19.7)	14(23.7)		82(20.1)	4(23.5)	

Abbreviations: UTUC= Upper tract urothelial carcinoma; LVI= lymphovascular invasion.

### Association with clinical and pathologic characteristics

The median preoperative pre-albumin, albumin was 27.2 mg/dL(IQR, 22.5-31.3) and 41.8 g/L (IQR, 39.6-44.1). Lower pre-albumin was significantly associated with older age, higher tumor stage (muscle invasive vs nonmuscle invasive), higher rate of diabetes, regional lymph node metastasis and LVI (all p<0.05**, Table 1), but not gender, tumor location and tumor grade. On the other hand, hypoalbuminemia was only significantly associated with diabetes (p<0.05**, Table 1).

### Association with prognosis of UTUC

**Figure 1:**
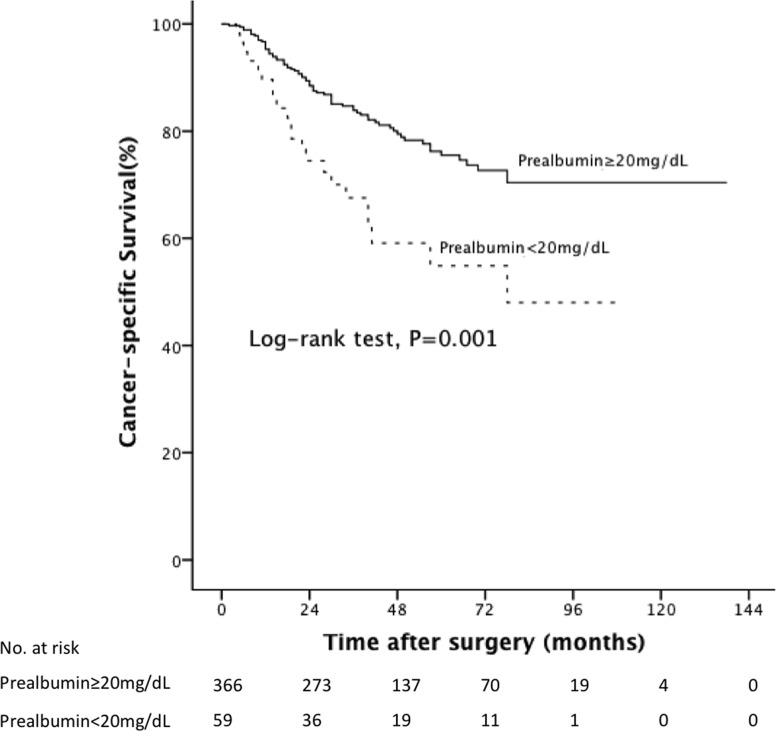
Kaplan-Meier curves predicting CSS by preoperative pre-albumin

**Figure 2:**
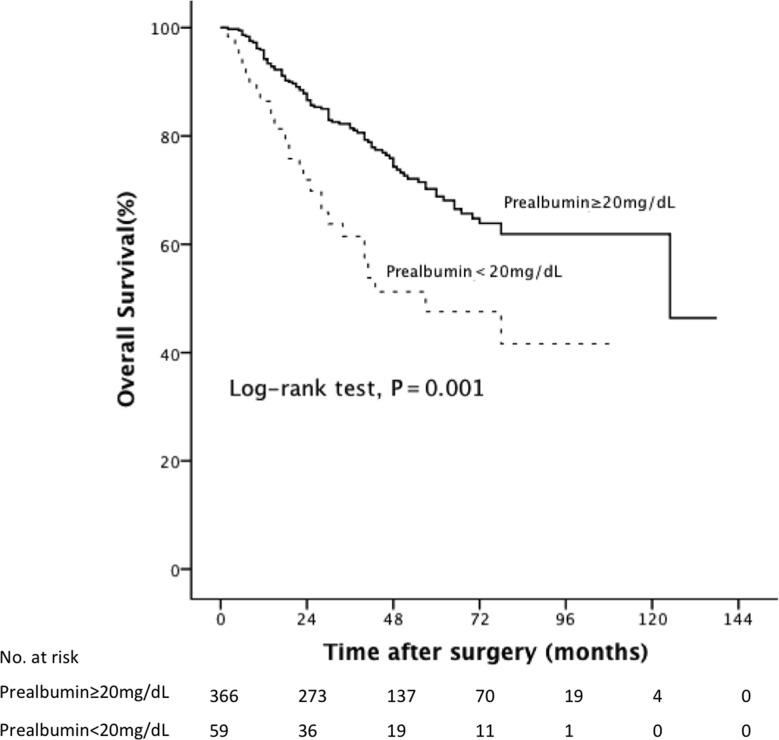
Kaplan-Meier curves predicting OS by preoperative pre-albumin

At a median follow-up period of 38.5 months (IQR, 23.0-62.0), low pre-albumin level was associated significantly with poor prognosis. In particular, the patients with preoperative low pre-albumin level had a significantly reduced rate of survival than those without low pre-albumin level with regard to CSS and OS (log-rank test, all P=0.001). The 5-year CSS rate was 54.9% in patients with low pre-albumin level and 75.5% in patients with normal pre-albumin level (Figure 1). Meanwhile the 5-year OS rate was 47.6% in patients with low pre-albumin level vs 68.8% in patients with normal pre-albumin level (Figure 2). Tables 2 and 3 show the results of the stepwise Cox survival analyses for predicting the CSS and OS, respectively. Multivariable analysis showed that low pre-albumin level (HR, 1.85;P=0.013) was an independent predictor of CSS, along with age greater than 65 (HR, 2.49; P<0.001), tumor multifocality (HR, 1.81; P=0.017), pT stage(HR, 3.08; P<0.001), grade(HR, 2.28; P=0.004), LVI(HR, 1.77;P=0.012), but hypoalbuminemia was not. As to OS, multivariable analysis also demonstrated that low pre-albumin level (HR, 1.73;P=0.015) was an independent predictor, along with age greater than 65 (HR, 3.03; P<0.001), tumor multifocality (HR, 1.85; P=0.007), pT stage (HR, 2.35; P<0.001), pN stage(HR, 1.94; P=0.029), grade(HR, 1.88; P=0.007), LVI(HR, 1.56;P=0.032), but hypoalbuminemia was not. The model with variables plus pre-albumin had higher c-index than the model with variables alone in CSS (0.775 vs. 0.771) and OS (0.765 vs. 0.760) in all patients.

**Table 2:** Univariable and multivariable Cox regression models to predict CSS in 425 patients treated with radical nephroureterectomy for UTUC

Variables	Univariable Analysis		Multvariable Analysis	
	HR(95%CI)	*P-*value	HR(95%CI)	*P-*value
Age greater than 65	2.41(1.52-3.83)	<0.001	2.49(1.56-3.97)	<0.001
Gender(Female vs. Male)	1.05(0.69-1.60)	0.828		
Hypertension(yes vs. no)	1.17(0.77-1.78)	0.468		
Diabetes mellitus (yes vs. no)	2.01(1.20-3.37)	0.008	-	-
Multifocality(yes vs no)	1.83(1.12-2.97)	0.015	1.81(1.11-2.96)	0.017
pTNM stage:				
T(2-4vs a-1)	4.36(2.70-7.02)	<0.001	3.08(1.88-5.07)	<0.001
N1-3 vs pNx/0	3.47(1.84-6.54)	<0.001	-	-
Grade (High vs Low)	3.48(2.03-5.96)	<0.001	2.28(1.30-4.02)	0.004
LVI(yes vs no)	2.79(1.81-4.30)	<0.001	1.77(1.13-2.76)	0.012
Adjuvant Chemotherapy (yes vs no)	1.64(1.03-2.61)	0.037	-	-
pre-Albumin(<20mg/dL vs≥20mg/dL)	2.18(1.35-3.51)	0.001	1.85(1.14-3.00)	0.013
Albumin(<35g/L vs≥35g/L)	2.51(1.22-5.18)	0.013	-	-

Abbreviations: HR= hazard ratio; CI=confidence interval; UTUC= Upper tract urothelial carcinoma; LVI= lymphovascular invasion.

**Table 3:** Univariable and multivariable Cox regression models to predict OS in 425 patients treated with radical nephroureterectomy for UTUC

Variables	Univariable Analysis		Multvariable Analysis	
	HR(95%CI)	*P-*value	HR(95%CI)	*P-*value
Age greater than 65	2.90(1.89-4.46)	<0.001	3.03(1.97-4.68)	<0.001
Gender(Female vs. Male)	0.95(0.65-1.39)	0.796		
Hypertension(yes vs. no)	1.11(0.76-1.61)	0.600		
Diabetes mellitus (yes vs. no)	2.19(1.40-3.44)	0.001	-	-
Multifocality(yes vs no)	1.71(1.10-2.65)	0.017	1.85(1.18-2.90)	0.007
pTNM stage:				
T(2-4vs a,1)	3.25(2.20-4.82)	<0.001	2.35(1.56-3.56)	<0.001
N1-3 vs pNx/0	3.59(2.05-6.30)	<0.001	1.94(1.07-3.53)	0.029
Grade (High vs Low)	2.65(1.71-4.10)	<0.001	1.88(1.19-2.99)	0.007
LVI(yes vs no)	2.38(1.60-3.53)	<0.001	1.56(1.04-2.35)	0.032
Adjuvant Chemotherapy (yes vs no)	1.28(0.82-1.98)	0.280		
Pre-Albumin(<20mg/dL vs≥20mg/dL)	2.08(1.36-3.20)	0.001	1.73(1.12-2.70)	0.015
Albumin(<35g/L vs≥35g/L)	1.96(0.96-4.01)	0.066		

Abbreviations: HR= hazard ratio; CI=confidence interval; UTUC= Upper tract urothelial carcinoma; LVI= lymphovascular invasion.

## DISCUSSION

In the present study, we investigated pre-albumin level, albumin level and other standard prognostic factors in 425 patients undergoing RNU for localized UTUC. Multivariate analysis showed that in addition to other well-established prognostic factors, preoperative pre-albumin level was an independent predictor of CSS and OS, but not hypoalbuminemia. To the best of our knowledge, this study was the first to investigate the prognostic impact of pre-albumin level in patients of urological cancer. Our study also showed that age greater than 65, tumor multifocality, pT stage, pN stage, grade, LVI were associated with survival outcomes, which is consistent with previous study [1].

Cancer-related malnutrition has a high incidence [15–17], and in patients with cancer, an impaired nutritional state has a negative impact on treatment outcomes, length of stay in hospital, prognosis and survival [9, 18]. It is well accepted that nutrition is an important determinant of immune responses and that malnutrition impairs the immune system, suppresses immune functions [19–22]. Furthermore, protein energy malnutrition is common in patients undergoing surgery and is associated with significant immunodeficiency, especially in the cell-mediated mechanisms, which is known to play a crucial role in the host defenses against infection or cancer [23].

Few studies have been conducted to determine the influence of perioperative nutritional status in the prognosis of UTUC patients [10, 24]. Kang et al. demonstrated that preoperative underweight (BMI<21.6 kg/m^2^) could significantly increase the rate of cancer-specific motility and overall mortality in UTUC patiets after RUN [24]. Ku et al. reported that preoperative hypoalbuminemia (albumin<3.5 g/dL) is an independent prognostic factor of CSS and OS in UTUC patients [10].

Pre-albumin is a visceral liver-synthesized protein, which is sensitive to determining malnutrition. Its biological half-life is approximately 2.5 days and is not altered by stress or acute inflammation [25]. Albumin has a half-life of 20 days and, as such, cannot be a suitable sensitive indicator of nutritional insufficiency [26]. So we hypothesize that the pre-albumin level may be a better indicator than albumin level for determining nutritional status and prognosis of UTUC. In our study, we found that in localized UTUC, the rate of low pre-albumin level (59/425,13.9%) was much higher than that of hypoalbuminemia (17/425, 4.0%). Also in the present series of patients with UTUC, those who had low pre-albumin level were more likely to have diabetes, higher T stage, N stage and lymphovascular invasion comparing with those with normal pre-albumin level. Meanwhile, hypoalbuminemia was only associated with diabetes in UTUC patients. These differences between patients with and without low pre-albumin level in terms of tumor characteristics may partly explain why the patients with low pre-albumin level in our cohort had more aggressive disease. In our multivariable analysis, low pre-albumin level was an independent predictor in UTUC patients but not hypoalbuminemia. The patients with low pre-albumin level had a 1.85-fold higher risk of dying of UTUC compared with those without low pre-albumin level.

Despite recent progress in the identification of genetic and molecular alterations in UTUC has been made [27], the most widely used routine prognostic assessment of UTUC currently still relies on traditional clinicopathological prognostic variables [28, 29]. The predictive accuracy of prognostic model might be improved by these biomarkers, but the high costs of analysis, the time-consuming preparation, and the lack of evidence together prevent them into clinical practice. In our study, we showed that adding pre-albumin was able to raise the predictive accuracy in this cohort of UTUC patients regarding to CSS and OS. The base model with traditional variables plus pre-albumin had higher c-index than the base model alone in CSS (0.775 vs. 0.771) and OS (0.765 vs. 0.760) in our cohort of patients. Because of the poor patient prognosis in UTUC, detailed understanding of individual risk factor associated with patients’ prognosis will impart benefits for outcome prediction and individual patient treatment choices, such as neoadjuvant chemotherapy and lymph node dissection. Thus we believe that the present results may add additional prognostic information to accurate assessment of individual risk profiles even before the surgery.

Limitations of this study include that the retrospective nature of the data collection and study completed at a single high-volume center. Additionally, we did not compare the pre-albumin with other nutritional tools.

## MATERIALS AND METHODS

### Patients

After institutional review board approval, the medical records from January 2003 to June 2013 archived at Renji Hospital were reviewed. During this period, 520 consecutive patients with nonmetastatic UTUC (Ta-4N0/+M0) underwent RNU at the department of urology at our institution. After excluding patients lost to follow-up within 3 months, patients without data on preoperative pre-albumin and albumin levels, patients with an active infection, patients with concomitant carcinoma invading bladder muscle and patients who underwent cisplatin-based neoadjuvant chemotherapy, there were 425 remaining patients who were included in the analyses.

Dissection of regional lymph nodes was performed in patients with lymph nodes enlarged in preoperative imaging. Pathological T stages were uniformly adjusted according to the 2009 TNM classification system [30] and tumor grade was assessed according to Tumor grading was assessed according to the 1998 WHO consensus classification [31]. We defined multifocal tumors as follows: the synchronous presence of two or more pathologically confirmed tumors in any location within the upper urinary tract [8]. Plasma pre-albumin and albumin levels were routinely measured 1 to 3 day before surgical intervention as a routine preoperative examination. Pre-albumin level was accessed by turbidimetric immuno-assay (Hitachi, Tokyo, Japan). The cutoff points to stratify pre-albumin and albumin were using pre-albumin <20 mg/dl to identify low pre-albumin, albumin <35 g/L to identify hypoalbuminemia [32]. AC would be offered as an option to patients with muscle invasive disease, lymph node positive disease, or pT1 disease with LVI, high grade disease or tumor multifocality after surgery. The patient would make a decision after the benefits and side effects of postoperative AC were explained by the treating urologist and oncologist together. The chemotherapy patients were administered 1000 mg/m2 gemcitabine on day 1, and 8 and 70 mg/m2 cisplatin on day 2 for the GC regimen. Cisplatin was replaced by carboplatin if the glomerular filtration rate was less than 40 ml/minute/1.73 m^2^. Four to 6 cycles of chemotherapy were planned according to patient status.

Patients were assessed by urine cytology and cystoscopy 3 months and every 6 months for 3 years after RNU and every 12 months thereafter. Computed tomography and/or magnetic resonance imaging were also performed every 6 months for 3 years and annually thereafter. CSS was defined as the time in months from the date of surgery to cancer related death. OS was defined as the time in months from the date of surgery to patient death from any cause.

The statistical software SPSS, v21.0 (IBM Corp., Armonk, NY, USA) was applied to all the analyses in this study. As to the clinicopathological outcomes according to the pre-albumin and albumin, for continuous variables, the Student t test was used for the variables reported as median (interquartile range (IQR)), for categorical variables, the chi-square and continuity corrected chi-square tests were used. The survival distributions were estimated by the Kaplan-Meier method and compared by a log-rank test. Univariable Cox proportional hazards models were used to address the relative impact of categorically coded low pre-albumin level, hypoalbuminemia, and other clinical and pathologic variables on CSS and OS. To prevent against overfitting by decreasing the number of variables, we selected only variables that were significant in univariable Cox models for the stepwise Cox regression analysis. Hazard ratio (HR) and 95% confidence interval (CI) were computed. The Harrell concordance index with variables only or combined pre-albumin data were used to evaluate the prognostic accuracy. All p values reported are two-sided, and p<0.05 was considered statistically significant.
